# Geospatial Tracking of a Rabies Outbreak in the Eastern Cape Province, South Africa, Using Molecular Data

**DOI:** 10.1155/tbed/2795613

**Published:** 2026-07-08

**Authors:** Mmantshuruge J. Miyen, Antoinette Van Schalkwyk, Matthijs F. Ravensberg, Jared Strydom, Jacqueline Weyer, Antoinette Grobbelaar, Veronique V. Dermauw, Bas B. Oude Munnink, Carmen W. E. Embregts, Corine H. GeurtsvanKessel, Resoketswe C. Moropeng, Claude T. Sabeta

**Affiliations:** ^1^ Agricultural Research Council - Onderstepoort Veterinary Institute (ARC-OVI), Onderstepoort, Pretoria, South Africa, arc.agric.za; ^2^ Faculty of Sciences, Department of Biomedical Science, Tshwane University of Technology, Arcadia Campus, Pretoria, South Africa, tut.ac.za; ^3^ Independent Researcher, Port Elizabeth, South Africa; ^4^ Consultant, Port Elizabeth, South Africa; ^5^ Centre for Emerging Zoonotic and Parasitic Diseases, National Institute for Communicable Diseases, National Health Laboratory Service, Johannesburg, South Africa, nhls.ac.za; ^6^ Faculty of Health Sciences, Department of Medical Virology, University of Pretoria, Pretoria, South Africa, up.ac.za; ^7^ Department of Virology, School of Pathology, University of Witwatersrand, Johannesburg, South Africa, wits.ac.za; ^8^ Department of Biomedical Sciences, Institute of Tropical Medicine, Antwerp, Belgium, itg.be; ^9^ Faculty of Veterinary Science, Department of Veterinary Tropical Diseases, University of Pretoria, Onderstepoort, Pretoria, South Africa, up.ac.za; ^10^ Department of Viroscience, Erasmus Medical Centre, Rotterdam, Netherlands, erasmusmc.nl

**Keywords:** dogs, domestic, Eastern Cape province, glycoprotein gene sequencing, lyssavirus, phylogenetic analysis, rabies, South Africa, wildlife

## Abstract

Rabies is a zoonotic disease known to humankind for millennia. Despite being preventable, rabies is still neglected and causes human fatalities, predominantly in resource‐limited areas in Africa and Asia. In South Africa, rabies is maintained in both domestic and wildlife reservoir hosts, with frequent cross‐species transmission. This study utilized retrospective epidemiological data from the Eastern Cape (EC) province of South Africa from November 2020 until December 2024, which included a prominent dog rabies outbreak. The majority of the rabies cases were reported in the Nelson Mandela Bay Municipality (NMBM). Subsequently, the study aimed to establish the origin of the outbreak and track its spread within the NMBM and the EC province, using descriptive summaries on epidemiological data gathered from 2596 cases, as well as partial glycoprotein gene sequence analyses generated from a panel of rabies viruses (RABVs), originating from rabies‐infected dog brain tissues (*n* = 102), human samples (*n* = 4), domestic cats (*n* = 3), and livestock (*n* = 5). Throughout the outbreak in the EC, dogs (*n* = 705) were the most commonly infected host species, highlighting the central role this domestic carnivore plays in rabies epizootiology in this country. The RABVs analyzed from this outbreak shared significant sequence homology of 99% mean sequence identity, suggesting a common progenitor and supporting the historical introduction of rabies into South Africa. Based on the sequence data, the RABVs from this outbreak were delineated into three distinct clusters, with the majority of the samples grouping within the recently described DD‐I and DD‐II clusters and a single sample cluster with wildlife samples in cluster BEF‐II. Cumulatively, our data suggest at least three independent introductions of the RABV infection into the dog populations of the NMBM between 2021 and 2024, with DD‐II subgroup C being the most dominant. The introduction of rabies into the study area may suggest a more complicated explanation linked to lapsed dog vaccinations during the outbreak period.

## 1. Introduction

Rabies is a neglected and fatal viral zoonosis that poses a public and veterinary health threat in low‐ and middle‐income countries (LMICs), including South Africa. The disease disproportionately affects LMICs, characterized by inadequate or nonexistent rabies control measures, which carry 99% of the global disease burden. Worldwide, it is estimated that at least 59,000 humans (95% CI: 25,000–159,000) succumb to the disease annually [1, 2], with 50% of these deaths reported in children under 15 years of age [3]. Rabies virus (RABV) is generally transmitted through the saliva of a rabid animal, often during bite contact and, in some cases, through scratches and mucous membranes, as well as organ transplantation. The disease is characterized by an acute, progressive, incurable viral encephalitis and has been reported in all mammalian species [4, 5].

The etiological agents of rabies are members of the *Lyssavirus* genus (family *Rhabdoviridae*, order *Mononegavirales*). RABV is the prototype member of the genus, given its huge public health and veterinary impact [6]. At present, 18 viral species in the genus are recognized by the International Committee on the Taxonomy of Viruses (ICTV) [7]. Lyssaviruses can be divided into at least three different phylogroups with distinct pathogenicity and immunogenicity [8]. Members of phylogroups 1 and 2 are both found in South Africa. Phylogroup 2 contains *Lyssavirus lagos* (LBV) and *Lyssavirus mokola* (MOKV), and both are exclusively of African origin [9]. Little or no cross‐protection with pre‐exposure prophylaxis (PrEP) or conventional rabies postexposure prophylaxis (PEP) was shown against lyssaviruses in phylogroup 2 [10, 11], and neither was there any cross‐neutralization across the phylogroups. Hence, current RABV vaccines may not provide adequate cross‐protection against all the genetically divergent lyssaviruses.

In South Africa, and within phylogroup I, there are two rabies biotypes independently maintained in the *Herpestidae* and *Canidae* families, respectively [12–15]. The former, initially known as *Viverridae* [16], and now referred to as the mongoose rabies biotype, is principally maintained by the yellow mongoose (*Cynictis penicillata*) on the highveld plateau of the country. Molecular clock analysis estimated the mongoose rabies biotype to be at least 200 years old, concurring with literature describing rabies in mongooses since the early 1800s [17], prior to canid rabies, and hence is thought to be indigenous to the region [12]. Canid rabies was for the first time introduced into the Gqeberha area (formerly Port Elizabeth) of the Eastern Cape (EC) province in 1893 through the importation of a rabid terrier dog from England [12]. The outbreak, associated with the terrier dog, was exclusively limited to domestic animal species and resulted in at least one human fatality [18]. The most recent introduction of canid rabies into the southern African region occurred in the late 1940s [12] and is believed to have been imported by dogs from Angola. Cross‐species transmissions of one rabies biotype into the host range of the other occur from time to time [19]. In this context, the canid rabies biotype successfully established cycles in wildlife carnivore species, including the black‐backed jackal (formerly *Canis mesomelas*) and now known as *Lupulella mesomelas* in Limpopo [20] and the North‐West provinces [21] and in bat‐eared foxes (*Otocyon megalotis*) in the Northern Cape Province of the country [22, 23]. Sporadic cases of rabies were encountered in the Limpopo province prior to 1974, mostly in jackals, to a lesser extent in cattle, and only occasionally in dogs and cats [24]. Recent scientific evidence and data support the aardwolf (*Proteles cristatus*) to be a new maintenance host of canid rabies in the Northern Cape province [25, 26].

The majority of animal and human rabies cases in South Africa have been reported from the KwaZulu/Natal province, where the disease has been maintained in dog populations since the late 1970s. Rural–urban migration coupled with the development of informal settlements associated with large dog populations capable of maintaining the disease has been the key drivers of rabies outbreaks in the province [27]. In 2021, a large dog rabies outbreak was reported in the Nelson Mandela Bay Municipality (NMBM) with about 705 confirmed dog rabies cases [28]. Due to control and preventive measures that were implemented in response to this outbreak, by 2024, rabies cases had declined drastically in the NMBM [29]. This study was designed to investigate the origin of the 2021 rabies outbreak in the NMBM and to track its spread within the metro and the EC province based on partial glycoprotein and the G‐L gene sequence analyses and spatial and temporal mapping. The genetic relationships of the RABVs from this outbreak were used to investigate how the virus spread throughout the dog population in the province, and the data could be used as a basis for future epidemiological studies. The model for public–private partnerships in the conduct of vaccination campaigns and rabies control as applied in the NMBM is useful to apply in the future as appropriate control measures to break the rabies transmission cycles and to mitigate potential human fatalities.

## 2. Materials and Methods

### 2.1. Study Area

**Figure 1:**
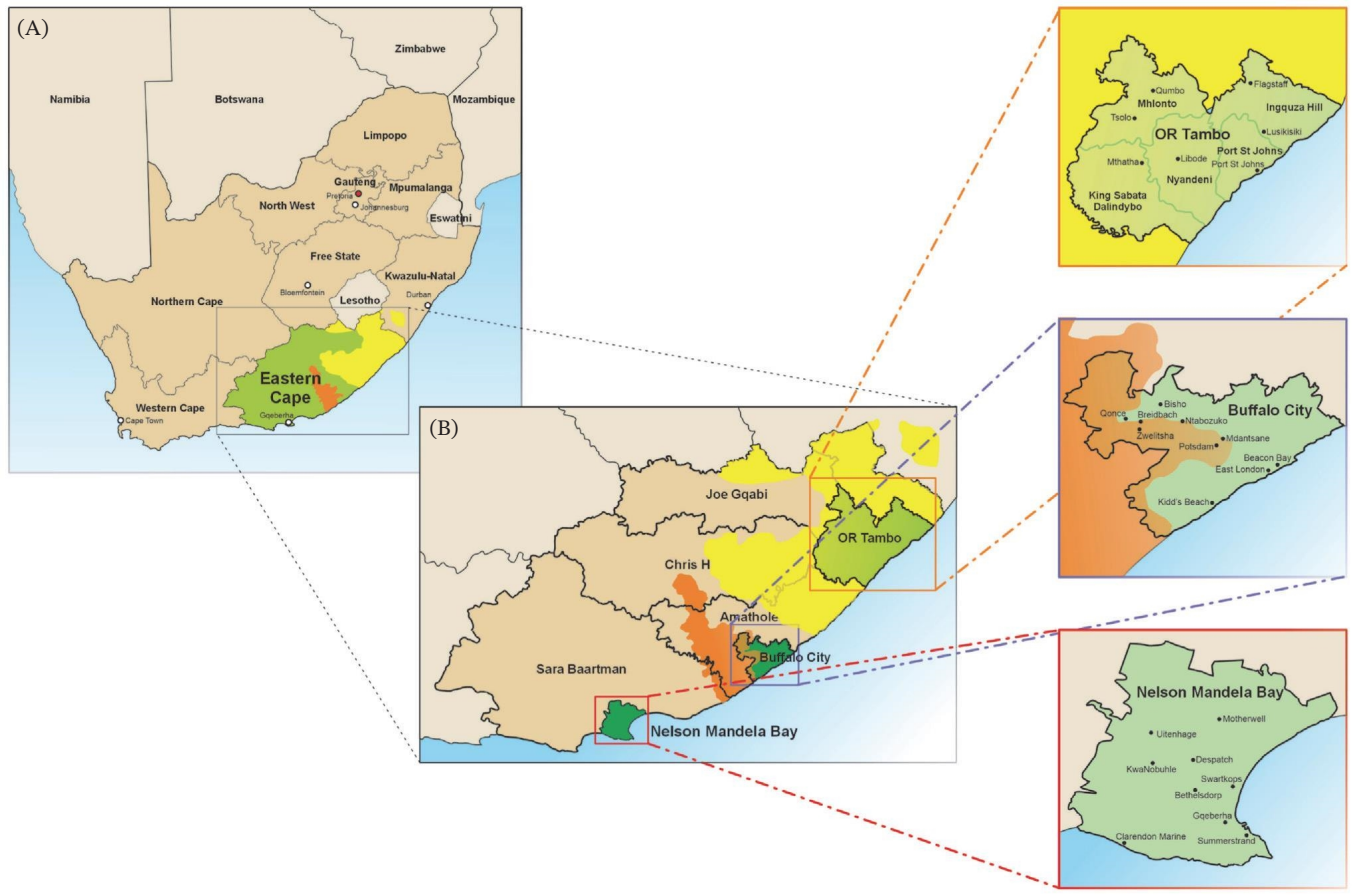
Map of South Africa indicating the Eastern Cape province in green, yellow, and brown (A). The yellow and brown shades represent the former Transkei and Ciskei homelands, respectively. (B) Indicates the EC with the dark green areas depicting the geographical areas affected by the dog rabies outbreak and the locality of origin of the samples analyzed in this study. Each of the green areas is individually indicated in three sections.

The EC province Figure 1A is located in the southeast of South Africa and is surrounded by the Indian Ocean, the Western Cape, Northern Cape, Free State, and KwaZulu/Natal provinces as well as the landlocked kingdom of Lesotho. The province spans a 168,966 km^2^ area and comprises six district municipalities (Sarah Baartman, Chris Hani, Joe Gqabi, Alfred Nzo, OR Tambo, and Amathole) and two metropolitan municipalities (NMBM and Buffalo City Metropolitan Municipality [BCMM) (Figure 1B). The six district municipalities consist of 31 local municipalities. Based on the 2022 census data (Statistics South Africa), the human population of the EC province is estimated at 7,230,204 [30], of which 1,190,496 people are in NMBM and 975,255 in BCMM. A large part of the EC province consists of the former homelands of Ciskei and Transkei (Figure 1B). These areas are remote and not easily accessible due to topography. The homelands are areas that have historically been impoverished and remain at high risk as transmission conduits of rabies from the KZN province. In addition, they have limited access to veterinary services, high stray dog populations, and inadequate vaccination coverages.

### 2.2. Outbreak Investigation

In order to describe the rabies outbreak, data on brain samples tested for RABV antigen were retrieved from the two laboratories approved for rabies testing in the country: the Allerton Provincial Laboratory (KwaZulu/Natal province) and the Agricultural Research Council–Onderstepoort Veterinary Institute (ARC‐OVI) (Gauteng province) (Figure 1A). The brain samples were collected from rabies‐suspect animals by state or private veterinarians and those based in non‐governmental organizations (NGOs) such as the Animal Welfare Society (AWS) and the Society for the Prevention of Cruelty to Animals (SPCA) (in Uitenhage) or the private sector. The samples were then submitted to the two diagnostic laboratories by the State Veterinary Services, where they were subjected for postmortem testing using the gold standard direct fluorescent antibody (DFA) test [31]. Test results for the rabies‐suspect animals for the period spanning November 2020 to December 2024 were included for analysis in this study. In addition to the DFA test results (positive/negative) obtained, the epidemiological information of the samples, including the collection date, location, and host species of origin, were also retrieved. In addition, human rabies case data were received from the National Institute for Communicable Diseases (NICD) of the National Health Laboratory Service, the only diagnostic facility authorized to test for human rabies in South Africa.

Additionally, dog vaccination records for the years 2021–2024 in NMBM were provided by the State Veterinary Services and South African Veterinary Association Community Veterinary Clinics (SAVA‐CVC). These records exclude data for routine vaccinations from the private veterinary clinics from NMBM.

### 2.3. Molecular Analyses

A total of 114 samples confirmed positive by DFA and originating from the study area were subjected to subsequent nucleotide sequence analyses. Only those samples that were available in the repositories at Allerton and ARC‐OVI diagnostic laboratories at the time of this study were included for sequence analysis. Specific details on the subset of RABVs from dogs and other host species are presented in Supporting Information 1: Table S1 (RABVs from EC province, South Africa, included in this study).

Viral RNA was extracted from both animal and human brain tissues using Tri‐Reagent (Sigma‐Aldrich) and the RNeasy lipid tissue mini kit (Qiagen, Germany), respectively, according to the instructions of the manufacturers. The RNA was used as a template in subsequent complementary DNA (cDNA) synthesis, as described previously [14]. Thereafter, a partial region of the cytoplasmic domain of the glycoprotein and the G‐L intergenic region of the rabies viral genome (a pseudogene) was amplified using the G (+) and L (−) primer set [32, 33]. For both the human and animal RNA samples, all the steps for cDNA synthesis were similar, except that the Transcriptor One‐step RT‐PCR kit (Roche Diagnostics, Germany) was used for human samples. The thermal cycling conditions for the amplifications were as follows: 94°C for 2 min; followed by 30 cycles of 94°C for 50 s, 45°C for 90 s, and 72°C for 60 s; and concluded with a final extension (72°C) for 7 min. The amplicons were separated and visualized in 1% ethidium‐bromide stained agarose gels and purified using spin columns (Zymo Research & Promega Corporation, USA), according to the manufacturers’ guidelines.

The purified amplicons were sequenced using Sanger dideoxy chain termination chemistry employing both the forward and reverse primers incorporated in the initial PCR (Inqaba Biotech, Pretoria, South Africa, and NICD, Johannesburg, South Africa) using the ABI Prism 3500XL Genetic Analyzer (Thermo Fisher, USA). The raw nucleotide sequence data were imported into CLC Genomic Workbench software (version 9), and single consensus sequences were assembled for each sample using the generated forward and reverse sequences. For the human samples, consensus sequences were generated using BioEdit Sequence Alignment Editor, version 7.7.1.

For whole genome sequencing (WGS), RNA was reverse transcribed using SuperScript IV (Invitrogen, USA) and random primers. dsDNA was generated using 3^′^–5^′^ Klenow DNA Polymerase (New England Biolabs, USA). The KAPA Hyper Plus kit (Roche, Switzerland) was used to prepare the library for sequencing. Some minor adjustments were made: The fragmentation time was reduced to 1 min, and the adapters were diluted to 1:100. Targeted enrichment was performed using VirCapSeq [34]. After a posthybridization PCR, samples were quantified, pooled equimolarly, and sequenced on an Illumina NextSeq (2 × 300 bp). Sequence reads were quality controlled using fastp and deduplicated using BBnorm, after which the sequences were assembled using SPAdes and the RABV sequences were extracted.

The consensus sequences, obtained from either full genome sequences of the field samples (Erasmus University, Netherlands) or partial glycoprotein gene sequences (ARC‐OVI and the NICD, Gauteng, South Africa), were further trimmed to 592‐nts encompassing the carboxyl domain of the glycoprotein and the G‐L intergenic region, which constitutes a highly variable region of the RABV genome. All the sequences in the alignment files (in ClustalW format) were submitted to the NCBI GenBank and allocated unique accession numbers (Supporting Information 1: Table S1).

### 2.4. Data Analysis

In order to elucidate the spatial and temporal evolution of the rabies outbreak, a base map was hand‐drawn, using as templates, maps from https://en.wikipedia.org/wiki/List_of_municipalities_in_the_Eastern_Cape and https://municipalities.co.za/provinces/view/1/eastern-cape. The resulting drawing was then scanned, colored, and modified using Adobe Photoshop Elements (Adobe Systems Inc., San Jose, CA, USA). A map showing all the confirmed rabies cases per local municipality was then created. Various graphs and tables were created using Excel.

Phylogenetic analysis was conducted using the RABV sequences from animal species from the EC province generated during the outbreak period (*n* = 114), human RABV sequences (*n* = 4), and, in addition, historically sequenced RABVs (*n* = 62) from the EC and KwaZulu/Natal provinces, as described previously [26]. The alignment files were used as input in phylogenetic tree reconstruction using the maximum likelihood (ML) method and the Kimura two‐parameter model (1980) for estimating genetic distances with 1000 bootstrap iterations in MEGA X [35].

For a more detailed temporal and spatial analyses of the outbreak, nucleotide sequences generated in this study with verified GPS coordinates (*n* = 91) were combined with historically sequenced viruses from 2003 (*n* = 9), 2010, and 2011 (*n* = 3) and a human sample from 2019, and then subjected to Bayesian analysis in SPREAD v1.0.7 [36]. In the initial genetic analysis, several RABVs from the NMBM were 100% sequence homologous and were therefore removed from further phylogenetic analysis. The 592‐nt sequences with sample collection dates and grid references were imported into BEAUti v1.10.4. The 100,000,000 iteration Markov Chain Monte Carlo (MCMC) analysis was performed using the HKY model, uncorrelated relaxed clock, and exponential growth tree selected as priors [37] in BEAST v1.10.4 [38]. The reliability of the results was scrutinized with Tracer v.1.7.1 (to check if ESS values were beyond threshold > 200), and a maximum clade credibility (MCC) tree was generated in TreeAnnotator v1.10.4. The newly generated MCC tree was visualized in Figtree v.1.4.2 and used in SPREAD v1.0.7. The output file from the latter was observed in Google Earth Pro.

## 3. Results

### 3.1. Laboratory Testing

Between November 2020 and December 2024, a total of 2596 samples were submitted from the EC province to both testing laboratories for rabies diagnosis (Allerton Provincial Laboratory and the ARC‐OVI [Onderstepoort]). Of the 2596 samples, 10 were unsuitable for testing and therefore excluded from the analysis. An additional 16 samples were excluded due to their locality of origin being outside the study area and another two because of the absence of geographical coordinates assigned to them (*n* = 28 samples in total excluded from testing). Of the remaining 2568 samples, 60.1% of the samples (*n* = 1544) were positive for rabies, with the majority originating from dogs (86.7%, *n* = 1339) (Table 1). Furthermore, of the total number of samples submitted between November 2020 and December 2024, 1256 (48.9%) were from NMBM, and 705 dog samples tested positive. Notably, during the height of the outbreak in NMBM (in 2021), 72% of the positive samples (*n* = 257) were collected through NGOs. Although the rabies outbreak in the EC province was largely dog‐driven, an additional 13 animal species were confirmed rabies positive, the vast majority being livestock (Table 2).

**Table 1:** Showing the distribution between positive and negative rabies cases in NMBM and BCMM.

Number of dogs and other animals that tested positive and negative	Total EC	NMBM	BCMM
Animals tested positive	1544	723	366
Number of dogs that tested positive	1339	705	339
Number of animals that tested negative	1024	533	172
Total	2568	1256	538

**Table 2:** Shows the summary of the number of positive rabies cases per species and spread over the municipalities.

Host species of origin	Number of positive rabies cases	Number of municipalities affected
Cat	20	9
Dog	1339	30
Total companion animals	1359	—
Cow	85	20
Donkey	3	1
Goat	35	13
Horse	8	5
Pig	10	8
Sheep	36	13
Total livestock	177	—
Aardwolf	2	2
Bat‐eared fox	1	1
Dassie	1	1
Genet	1	1
Mongoose	1	1
Polecat	2	2
Total wildlife	8	—

### 3.2. Outbreak Description

**Figure 2:**
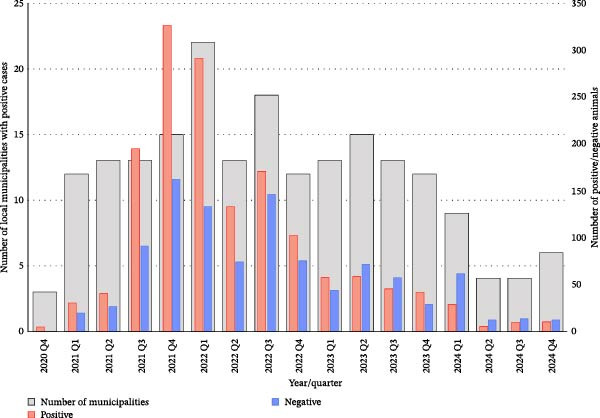
A graph showing the overall trends of positive and negative rabies cases in the local municipalities of the Eastern Cape province, 2020–2024.

**Figure 3:**
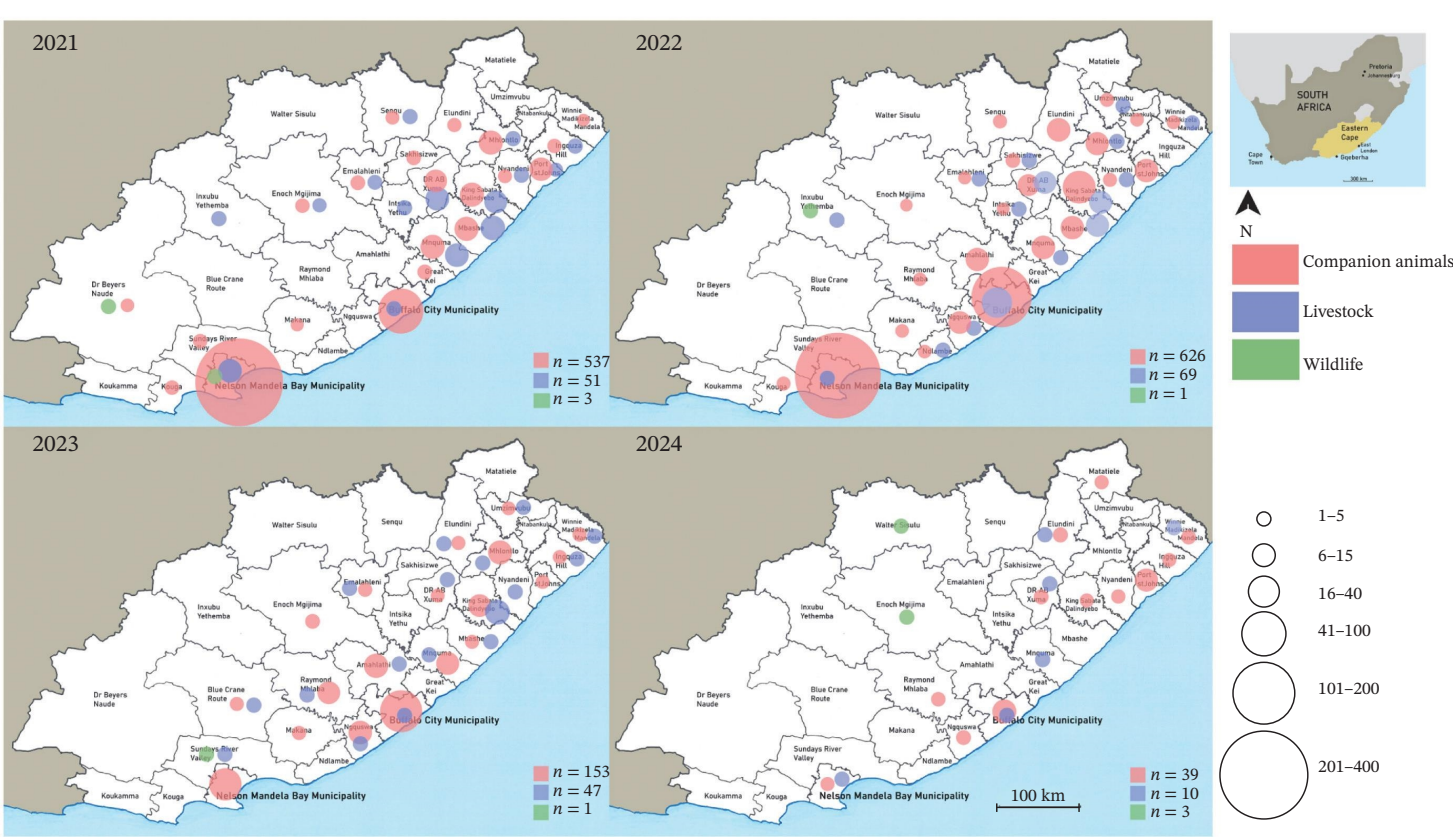
A panel of images showing spatial distribution of different animal species that tested positive for rabies per municipality, Eastern Cape province, for the years 2021–2024.

**Figure 4:**
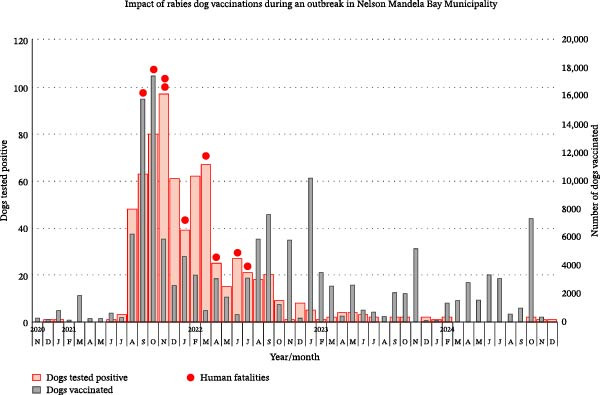
A graph showing the impact of vaccination on numbers of dog rabies positive cases and human deaths in NMBM from November 2020 to December 2024.

In the last quarter of 2020, the EC province recorded four positive rabies cases in animals spread over three municipalities. Notably, one of these was a domestic dog (LIMS 2021010022) from Bluewater Bay, an area located in the eastern part of the NMBM and, interestingly, a municipality with no previous record of positive rabies cases. Another dog from the same area tested positive in the first quarter of 2021, and 30 animals were confirmed positive from 12 different municipalities during the same period (Figure 2). On 16 June 2021, rabies was confirmed in another domestic dog in Gqeberha, the biggest city in the EC province. By the end of July 2021, a drastic increase in the number of rabid dogs was observed in this metropolitan area, and positive rabies cases were simultaneously detected in the BCMM (Figures 3 and 4). Animal rabies positive cases peaked at 326 in the last quarter of 2021, and the spread of rabies reached a maximum, with 22 municipalities involved in the first quarter of 2022. Starting from the third quarter of 2022, right through to the fourth quarter of 2024, the number of rabies cases declined drastically from 170 to 12. The geographical spread, averaging 11.6 municipalities with positive rabies cases per quarter, also diminished to five in the last quarter of 2024 (Figure 2). A high number of animal rabies cases confirmed during the peak of the outbreak originated from the NMBM and, to a lesser degree, the BCMM (Figure 3). In the two metropolitan areas, the number of dogs tested positive: Livestock tested positive (DL) ratios were 70:1 and 15:1 for NMBM and BCMM, respectively (see Figure 3). This is in contrast to the rural eastern municipalities, where the DL ratio on average was 2:1. Throughout this rabies outbreak, all, with the exception of one municipality of KouKamma (Figure 3), recorded positive rabies cases in animals. Wildlife rabies cases were mostly observed in the dry north‐eastern part of the province (Figure 3). For the whole province, 25 humans were confirmed to have succumbed to rabies, and another six cases were probable. Of these, six were adults, two were teenagers, and 14 were children. For the remaining cases, no demographic data were available. All human rabies cases were associated with dog bites.

### 3.3. Vaccination Campaign

A state vaccination campaign, supported by NGOs and private veterinary practices, was initiated in August 2021, reaching 209 sites within the NMB municipality and vaccinating 55,901 dogs. In 2022, 212 sites were visited and 43,535 dogs vaccinated; in 2023, 119 sites and 20,419 vaccinations. The mass dog vaccination campaigns curbed rabies‐positive numbers but failed to eliminate the disease from the NMBM area, based on the few dog‐positive cases that were laboratory confirmed towards the end of 2024 (Figure 4).

**Figure 5:**
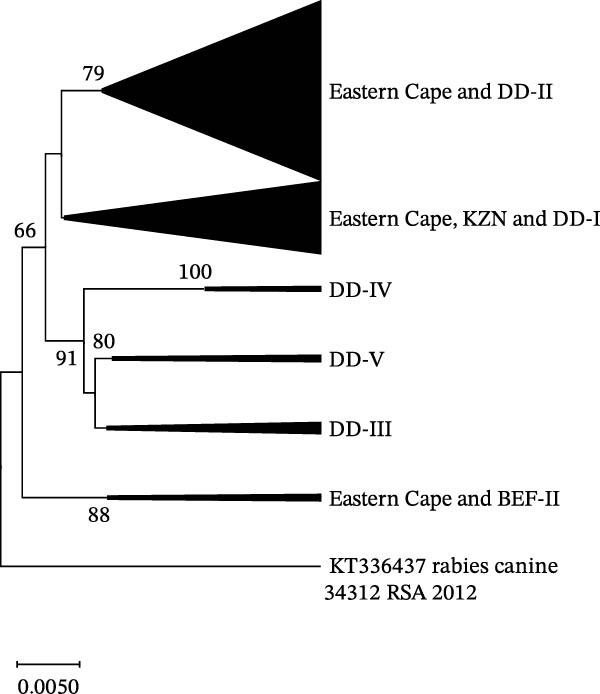
The phylogenetic tree was constructed using the maximum likelihood method for phylogenetic inference based on the Kimura two‐parameter model and applied to the 592‐nt region of the glycoprotein gene and the adjacent G‐L intergenic region of 176 rabies viruses, with a statistical frequency set as 1000 iterations. Lineages were identified as previously described [26]. The outlier is a dog RABV (Lab. reference # 343/12), confirmed in 2012 (KT336437).

Partial sequences of individual RABVs were generated from a panel composed of RABVs from domestic dogs (*n* = 104), domestic cats (*n* = 3), humans (*n* = 4), sheep (*n* = 2), and a pig (*n* = 1). From the ML tree (Figure 5), the RABVs from the 2021/2023 EC outbreak (*n* = 114) and other previously sequenced RABVs from KwaZulu/Natal and the EC province (*n* = 62) included in the final analysis shared significant sequence homology, with a mean sequence identity of 99% (Kimura two‐parameter model, mean pairwise distance matrix not shown). Phylogenetic analyses indicated that the newly generated sequences from the EC could be grouped within three previously described clusters: DD‐I, DD‐II, and BEF‐II (Figure 5).

**Figure 6:**
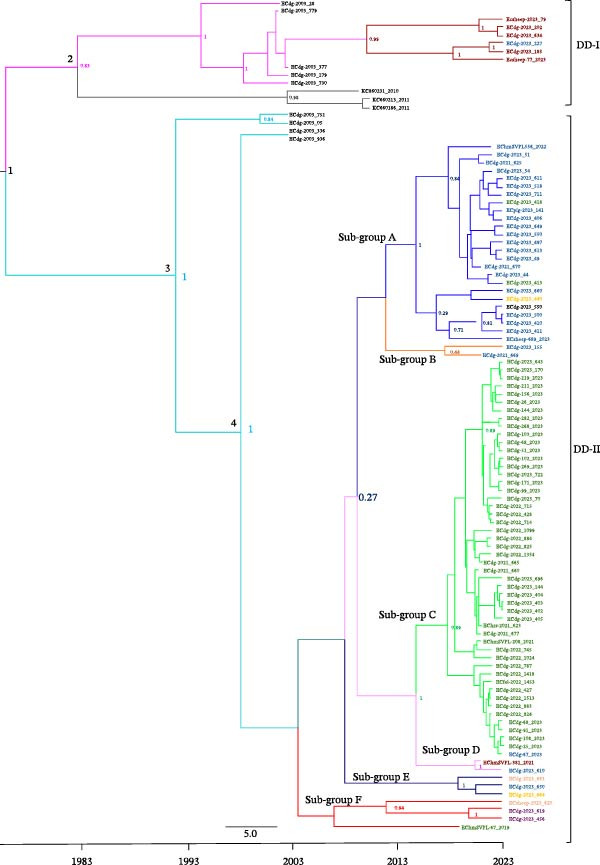
Bayesian phylogenetic analyses of a subset of the nucleotide sequences generated in this study (*n* = 91), as well as 12 sequences from outbreaks in 2003, 2010, and 2011 belonging to clusters DD‐1 and DD‐II. Four important nodes are indicated in the phylogenetic tree to highlight the divergence between DD‐I and DD‐II (1), within DD‐I (2), and within DD‐II (3 and 4). Six subgroups (A to F) are indicated in DD‐II. The names of the taxa were written in different colors to indicate their municipality of origin (NMBM are green, BCM are blue, OR Tambo are dark red, Elundi are orange, Encqobo are plum, Emalahleni are tan, and Raymond Mhlaba are purple).

The spatial and temporal distribution of a subset of the newly generated sequences (*n* = 91) was subjected to a Bayesian analysis. The DD‐I and DD‐II common ancestor is indicated as node 1 (Figure 6). It was evident that the sequences from RABVs confirmed in 2023 and belonging to DD‐I (Figure 6, red) shared a common and recent ancestor with the sequences obtained from RABVs in domestic dogs (in 2003) in the EC province (Figure 6). This 2003/2023 cluster shared a common, but not recent, ancestor (Figure 5, node 2) with the 2011 samples from KZN (Figure 6, gray). The 2023 sequences in cluster DD‐I originated from the OR Tambo municipality and were obtained from both dogs and sheep (Figure 6 and Supporting Information 2: Figure S1A). The clustering of samples from the EC and KZN provinces within DD‐I indicated that the 2003 and 2023 RABVs were genetically similar and belonged to the same lineage (unlike those from 2011), supporting the re‐emergence of rabies in this geographical area (Figure 6 and Supporting Information 2: Figure S1A).

In contrast, the sequences grouped within DD‐II were subdivided into six subgroups (A to F) (Figure 6), which share a common but not recent ancestor with the 2003 EC samples in DD‐II (Figure 6, light blue; nodes 3 and 4). The locality of origin of the 2003 samples is indicated in Supporting Information 3: Figure S1B, while DD‐II is represented in Supporting Information 4: Figure S1C. Sequences belonging to subgroup A (Figure 6, blue) were submitted from the BCMM, located on the eastern seaboard of the EC province (Figure 6, blue lines). The samples were submitted between 2021 and 2023, indicating that the RABV had been circulating in dog populations in the area for at least a year and affected mainly domestic dogs but also humans and sheep. Subgroup B (Figure 6, orange) consisted of two RABVs associated with dogs from the NMBM in 2021 and 2023 (Figure 6, orange lines).

The majority of the samples analyzed in this study were submitted from the NMBM and formed part of subgroup C (Figure 6, green) characterized by localized outbreaks and a RABV from a human who succumbed to rabies in 2021. The majority of the virus sequences were obtained from dogs. The RABVs belonging to this subgroup were circulating in the NMBM during the 2021–2023 of the outbreak years (Figure 6, green lines). Two sequences belonging to subgroup D (Figure 6, pink) were obtained from a human sample and a dog in 2021 and 2023, respectively. Both samples in subgroup D originated from the BCMM (Figure 6). Three sequences in subgroup E (Figure 6, dark blue) originated from dogs in the OR Tambo district municipality in the north‐eastern part of the EC province, as well as in BCMM (Figure 6). Subgroup F (Figure 6, dark red) consisted of a virus obtained from a human sample in 2019 in the NMBM, while the remainder of the viral sequences were from dogs and a sheep in the BCMM (Figure 6, dark red lines).

## 4. Discussion

This study aimed to elucidate the transmission dynamics of dog rabies in the EC province during the major rabies outbreak of 2021. In addition, we wanted to establish the origin of the dog rabies outbreak and track its spread in the NMBM, the focal point of this dog outbreak. All the RABVs were found to be closely related (99% mean sequence identity), supporting a common progenitor and were epidemiologically linked to RABVs from KZN, lending support to findings from a previous research study [27], and consistent with the recent historical introduction of dog rabies cycles into southern Africa in the late 1940s [12, 27, 39]. The RABVs were grouped in either of the previously described clusters (DD‐I or DD‐II and a single sequence in BEF‐II) [26, 27]. The sequence data generated through the most complete molecular description of the canid rabies outbreaks in the EC and KZN provinces were the foundation for our present epidemiological investigation [26, 40]. The first rabies outbreak in the EC province described in history was reported in 1893 [12]. The second rabies outbreak started in the mid‐1970s and is known to have spread southwards into the EC province from KZN province (Figure 1). A decade later (in 1986), dog rabies reached the EC province was confirmed in the northern areas of the Transkei [41] and continued to spread throughout Transkei such that by the early 1990s, rabies had reached East London (BCMM, Figure 1). From 2008 to 2009, rabies re‐emerged in the northern areas of Transkei and was associated with many human fatalities, apparently more than any other province in the country.

**Figure 7:**
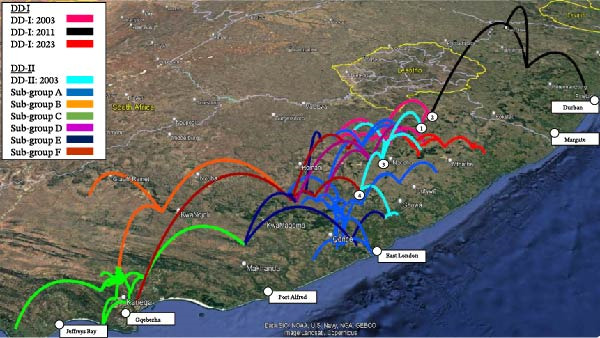
Spatial distribution of the rabies viruses in correlation with the Bayesian phylogenetic analyses performed in Figure 6, using Google Earth Pro. Four important nodes depicted in Figure 7 are indicated with white circles. The location of each sample is graphically represented on the map and at the end of the line, while the nodes are indicated as deflections. The different subgroups delineated in Figure 6 are depicted in corresponding colors.

While historically, the former Transkei and Ciskei regions of the EC province (now OR TAMBO and Amathole, respectively, Figure 1) have encountered rabies outbreaks for decades, dating back to the 1970s [12], the biggest city in the province, Gqeberha, and part of the NMBM and the surrounding areas, experienced its first large dog rabies outbreak in July 2021 [28]. Our genetic data support at least three separate introductions of RABV infection into the dog population of the NMBM, with the oldest one occurring about 2020 and coinciding with the historical observation of this outbreak. This may suggest a more complicated and interesting explanation that is linked to lapsed dog vaccinations during the outbreak. The introduction of the RABV infection was followed by local transmissions and significant diversifications, leading to widespread spatial distribution in the NMBM (Figure 7, Supporting Information 4: Figure S1C). In our initial genetic analysis, we found several RABVs from the NMBM with a 100% sequence homology, and some of these nucleotide sequences were removed from further phylogenetic analysis for a better resolution. The 100% sequence homology of some RABVs highlights and confirms the highly opportunistic nature of the dog rabies variant within the NMBM dog population available and ecologically capable of sustaining prolonged rabies cycles [39]. The localized phylogeographic patterns within the NMBM further demonstrate the role of constrained virus dispersal that is defined and shaped by regional transmission dynamics and limited movement of RABV‐infected dogs. A previously sequenced dog RABV confirmed during the 2010 rabies outbreak in Gauteng province [42] clustered with RABVs (DD‐1) from the EC province, highlighting the importance of human movement in rabies transmission events, for instance, from OR TAMBO to Gqeberha.

Spillover of RABV infections also known as cross‐species transmissions occur from time to time [19]. Our study demonstrated spillover of the dog rabies variant into companion animals, livestock, and some wildlife species (Supporting Information 6: Figure S3). Whereas the majority of the nondog host species analyzed were of the canid rabies variant, RABV originating from a mongoose and a domestic cat were shown to belong to the mongoose rabies biotype, supporting the notion that wildlife rabies is not of huge public health significance in this part of the country. The dog:livestock ratios obtained for the two metropolitan areas (of NMBM and BCMM), in comparison to the rural areas, support previous findings that the majority of livestock is found in the communal farming sector of the EC province, where a rabies resurgence was experienced. In this context, significant economic losses are incurred by communal farmers in the EC province as a result of rabies‐associated livestock mortalities; however, the full extent of this economic impact has not yet been comprehensively quantified.

Previous molecular epidemiological studies on RABV on the eastern seaboard of this country identified circulating lineages and reported that the dominance of the DD‐1 cluster targeted the partial glycoprotein and the G‐L gene region [26, 27, 40]. The findings from the time series Bayesian analysis supported the emergence of the DD‐II subgroup C lineage (in the NMBM), enhancing our understanding of rabies epidemiology and transmission dynamics in specific geographical municipalities in the province and underscoring the value of genomic surveillance in guiding targeted and evidence‐based control strategies that are aligned with the global goal of eliminating dog‐mediated human rabies by 2030. More specifically, our study allowed for a closer insight into the geographic spread of RABVs and lineage diversification in the EC province. It was interesting to note that the subgroups delineated during our analysis did not always remain localized to particular geographical areas, but their dispersal was often influenced by human movements [42], leading to the introduction of some lineages into new areas and their subsequent cocirculation. Overall, integrating genomic data with epidemiological data from the EC rabies cases (2020–2024) (Figure 3) further improved our understanding on the spread of rabies cases and identified possible points of introduction, which were at minimum three in the case of NMBM. In addition, the epidemiological and sequence data analyses suggest an urgent need for preventive vaccination, particularly targeting dogs in source endemic areas to mitigate livestock and human fatalities. The utilization of WGS compared to partial genome sequencing of RABVs and other viral and bacterial pathogens is now commonplace in several global regions and apparently gives a higher resolution. If we had applied WGS to this study, it is possible that we could have probably observed significant insights into the RABV transmission dynamics at scales that are relevant to local municipality control.

The lack of dog population data in the NMBM is an obvious limitation to rabies control. Hence, we could not ascertain the exact vaccination coverage achieved during the mass dog vaccination campaigns. In addition, dog vaccination data were only available for the NMBM but not for BCMM (where we observed a resurgence of the disease) and the rest of the EC province. The size of the dog populations in NMBM and in other parts of the EC province is unknown, but from personal communication with the state veterinarian, the dog population in NMBM is thought to be about 100,000. The state normally conducts dog vaccination campaigns in NMBM twice a year, but the 2021 cycle was disrupted due to the COVID‐19 pandemic, a situation shown to lead to increased incidence of rabies. This may explain the emergence and the rapid spread of the disease observed during the early part of 2021 [42]. The key objective of a successful canine rabies elimination program is to maintain a high enough level of rabies vaccination coverage to interrupt rabies transmissions within a defined dog population. This in turn reduces the incidence of rabies among human populations. South Africa has a strategy for the elimination of dog‐mediated human rabies in place (https://www.nda.gov.za/index.php/publication/425-animal-health-information).

Eliminating RABV infection from dog populations (i.e., the source) is still the most cost‐effective and reliable preventive measure for rabies, although it is underprioritized in most LMICs because of limited resources as well as competing veterinary and health priorities. Dog vaccinations have been shown to drastically reduce the demand for the use of the costly PEP in humans [43, 44]. For example, a vaccination campaign with a 50% coverage can lead to a breakdown of the rabies transmission cycle but may not necessarily lead to a complete elimination of the disease [44], hence the observed re‐emergence of the disease in the LMICs. The most recent city‐wide mass dog vaccination in NMBM is a good example and model of public–private partnerships, leading to the eventual control of rabies in the municipality [45]. These partnerships generally demonstrate a synergistic approach in the interruption of dog rabies transmission cycles and further highlight the need for enhanced communication between the relevant stakeholders of a One Health approach to rabies control [45]. Furthermore, partnerships on local and international levels are crucial for disease control [46–48]. For instance, in this study, state institutions and NGOs were pivotal in the collection and submission of central nervous tissue samples for rabies testing. In particular, the straw method was used extensively to collect brain tissue samples and avoid the usual delays experienced when sending whole carcasses to veterinary diagnostic laboratories.

Epidemiological surveillance for rabies involves the key epidemiological indicators, namely, (i) the number of rabies outbreaks observed/recorded, (ii) the total number of diagnostic tests performed, and (iii) trends of the positivity ratios (Supporting Information 5: Figure S2). In this study, the positivity ratios determined for each municipality per quarter appeared to suggest an improvement in surveillance activities, particularly towards the end of the outbreak period. However, the apparent improvement observed could also suggest or confirm the effect of mass dog vaccinations implemented in the municipality. In this study, state institutions and NGOs collected and submitted central nervous tissue samples for rabies testing using the straw method and circumvented the usual delays experienced when sending whole animal carcasses to veterinary diagnostic laboratories.

In conclusion, surveillance is key to any successful disease control program and facilitates identifying the rabies hotspots and spread of the disease within a specified geographical region. If done in real‐time, then timely intervention can be applied. Surveillance activities should ideally be supported by genetic sequencing of RABVs to infer the source of infection, given that genetic variants of the RABV are associated with specific rabies cycles and species of terrestrial carnivores in South Africa. Surveillance activities as described in this manuscript have recently supported identifying the emergence of a rabies cycle in a marine mammal, the Cape fur seals, on the western coastline of South Africa [49]. The successful control of the rabies outbreak described in this manuscript relies solely on vaccination and reaching 70% herd immunity but more importantly is driven by the multisectoral collaboration of the One Health Platform in the province, and this should be the foundation of the control of rabies and other zoonotic diseases in other provinces in South Africa.

## Funding

This work was supported by the Belgian Directorate‐General for Development Cooperation (DGD) within the DGD‐Institute of Tropical Medicine (ITM) Framework Agreement 5 (2022–2026).

## Ethics Statement

Ethical approvals for the research study were granted by the ARC‐OVI Animal Ethics Committee (AEC 23_03) and ARC‐OVI Biosafety and Biosecurity (IBBC 23.03). An additional approval was granted by the Faculty Committee for Research Ethics‐Science of the Tshwane University of Technology, certificate number FCRE 2023/05/009 (SCI) (FCPS 01). In addition, the section 20 approval was granted by the Director of Animal Health of the Department of Agriculture (DoA) under certificate number 12/11/1/1 (a) (2748KL). Approval for the collection and reporting of data related to human rabies cases in South Africa was provided for in the protocol entitled: Essential communicable disease surveillance and outbreak investigation activities of the NICD (Reference Number M210752, the Human Research Ethics Committee of the University of the Witwatersrand).

## Conflicts of Interest

The authors declare no conflicts of interest.

## Supporting Information

Additional supporting information can be found online in the Supporting Information section.

## Supporting information


**Supporting Information 1** Table S1: Rabies viruses originating from Eastern Cape province, South Africa, and included in this study.

**Supporting Information 2** Figure S1A: Distribution of samples belonging to DD‐I with the phylogenetic nodes 1 and 2 indicated with white circles.

**Supporting Information 3** Figure S1B: Geographical distribution of the DD‐II samples from 2003, with the phylogenetic nodes 1, 3, and 4 indicated with white circles.

**Supporting Information 4** Figure S1C: Geographical distribution of samples belonging to DD‐II, with the phylogenetic nodes 1, 3, and 4 indicated with white circles.

**Supporting Information 5** Figure S2: Overall trends of the positivity ratios of animal rabies diagnoses in the various municipalities of the Eastern Cape province, 2020–2024.

**Supporting Information 6** Figure S3: The evolutionary history was inferred by using the maximum likelihood method and Kimura 2‐parameter model. This analysis involved 38 nucleotide sequences.

## Data Availability

All genome sequences and associated metadata used in this study are available. The accession numbers, virus names, collection dates, originating and submitting laboratories, and contributing authors for each sequence visit the Genbank for specific epidemiological details.
