# Supplementary figures and images for: Geospatial Tracking of a Rabies Outbreak in the Eastern Cape Province, South Africa, Using Molecular Data
Source: Transbound Emerg Dis. 2026 Jul 8;2026:2795613. doi: 10.1155/tbed/2795613 (PMC13346776; doi:10.1155/tbed/2795613)

## Slide 1
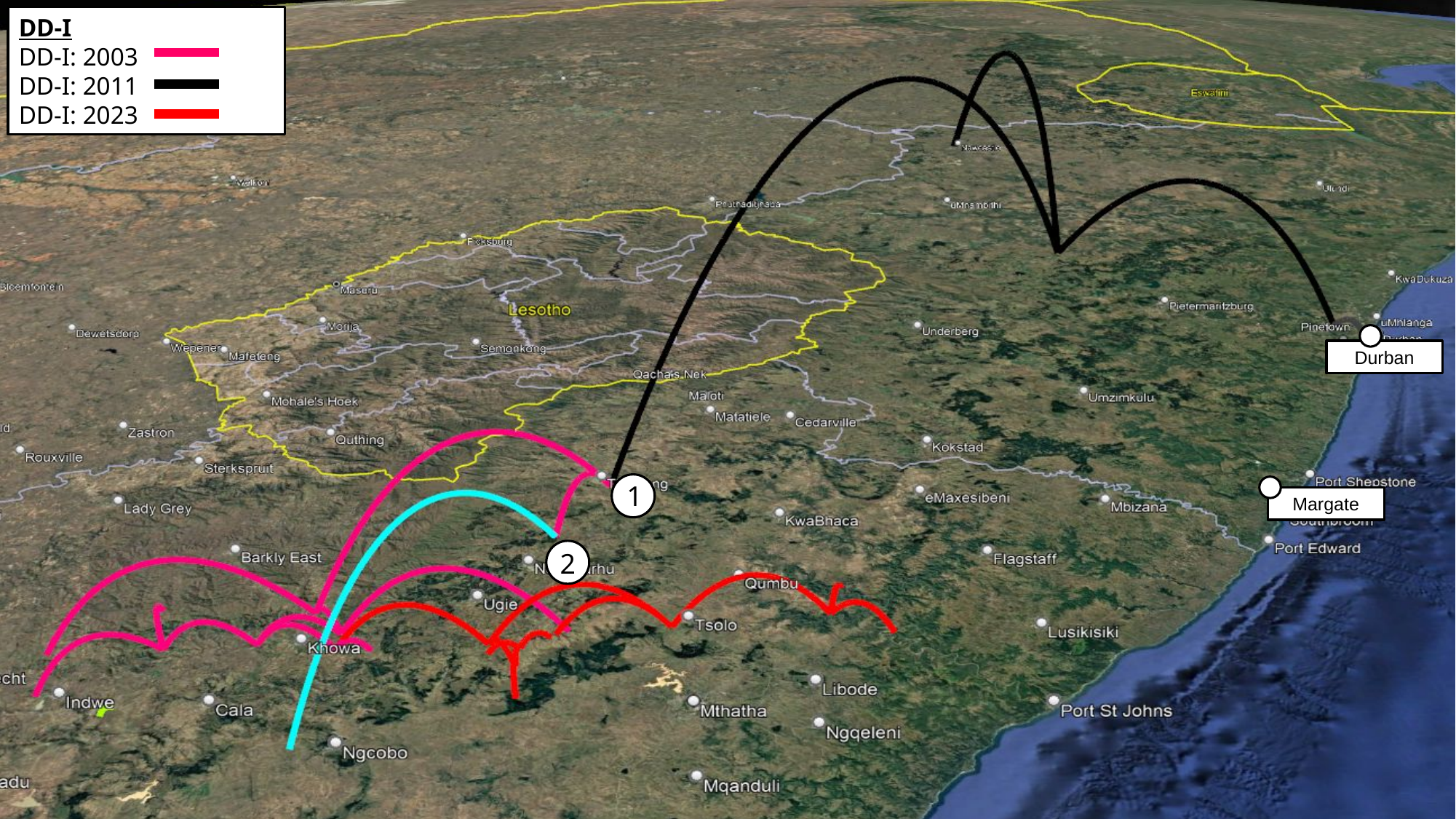

DD-I
DD-I: 2003
DD-I: 2011
DD-I: 2023
Durban
1
Margate
2

Supplement: Supplementary file 2 — Supporting Information 2 Figure S1A: Distribution of samples belonging to DD‐I with the phylogenetic nodes 1 and 2 indicated with white circles. [file TBED-2026-2795613-s004.pptx]

## Slide 1
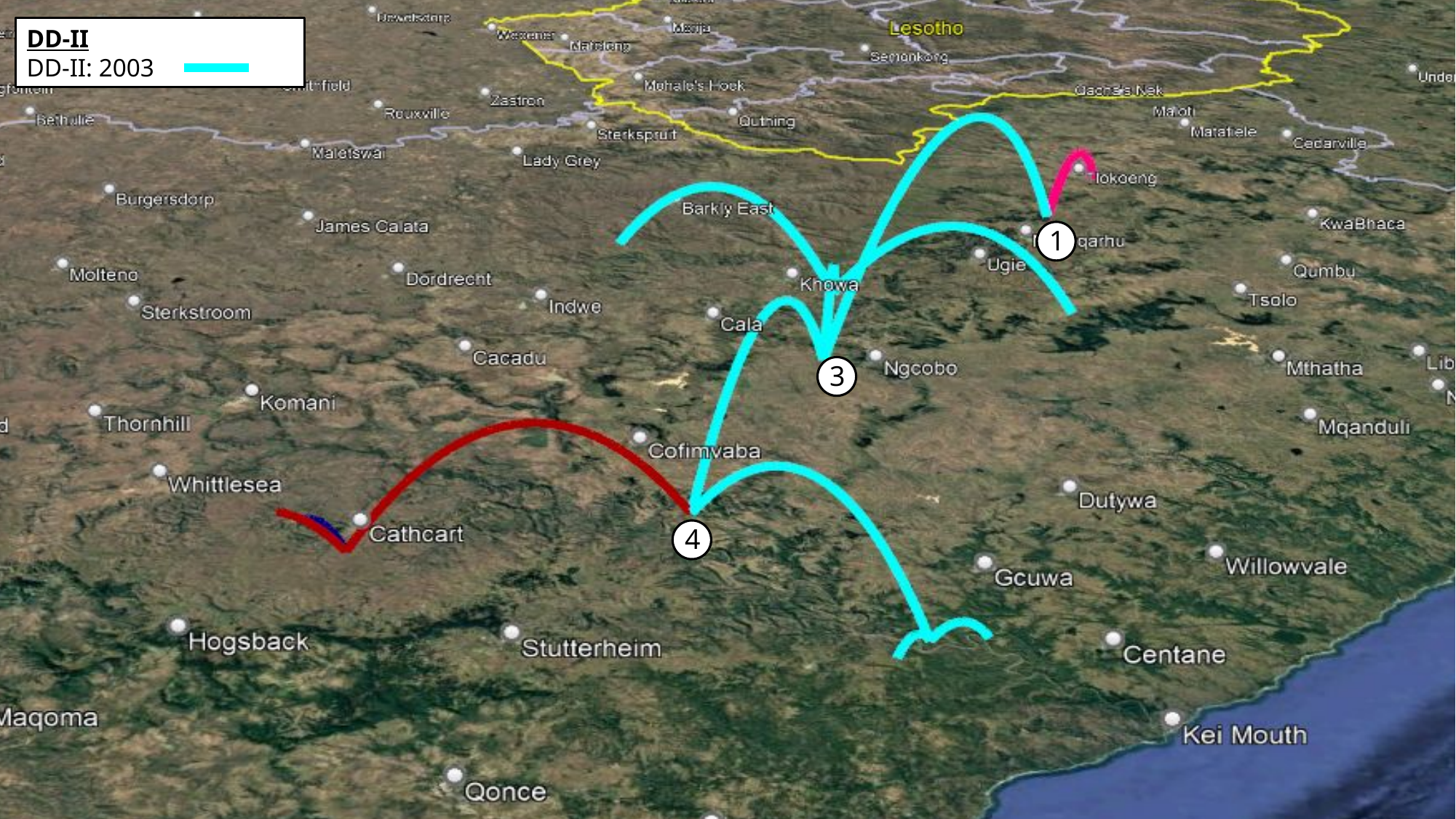

DD-II
DD-II: 2003
#
1
3
4

Supplement: Supplementary file 3 — Supporting Information 3 Figure S1B: Geographical distribution of the DD‐II samples from 2003, with the phylogenetic nodes 1, 3, and 4 indicated with white circles. [file TBED-2026-2795613-s003.pptx]

## Slide 1
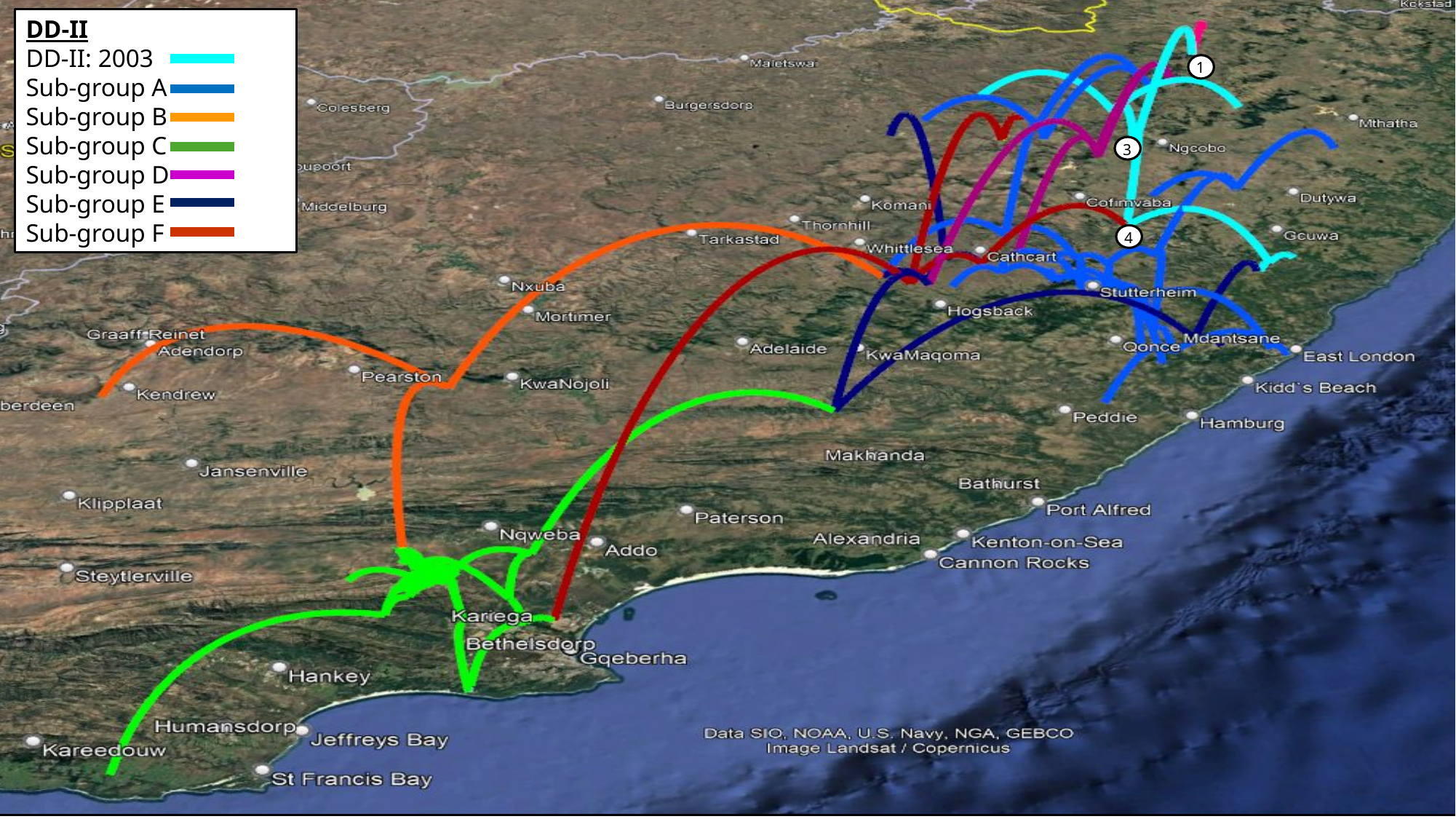

DD-II
DD-II: 2003
Sub-group A
Sub-group B
Sub-group C
Sub-group D
Sub-group E
Sub-group F
1
3
4

Supplement: Supplementary file 4 — Supporting Information 4 Figure S1C: Geographical distribution of samples belonging to DD‐II, with the phylogenetic nodes 1, 3, and 4 indicated with white circles. [file TBED-2026-2795613-s002.pptx]

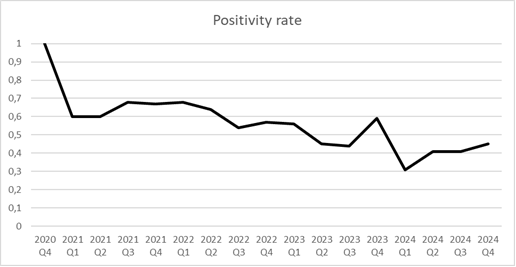

Supplement: Supplementary file 5 — Supporting Information 5 Figure S2: Overall trends of the positivity ratios of animal rabies diagnoses in the various municipalities of the Eastern Cape province, 2020–2024. [file TBED-2026-2795613-s001.docx]
