# Supplementary material for: Geospatial Tracking of a Rabies Outbreak in the Eastern Cape Province, South Africa, Using Molecular Data
Source: Transbound Emerg Dis. 2026 Jul 8;2026:2795613. doi: 10.1155/tbed/2795613 (PMC13346776; doi:10.1155/tbed/2795613)
Supplement: Supplementary file 6 — Supporting Information 6 Figure S3: The evolutionary history was inferred by using the maximum likelihood method and Kimura 2‐parameter model. This analysis involved 38 nucleotide sequences. [file TBED-2026-2795613-s006.pptx]

## Slide 1
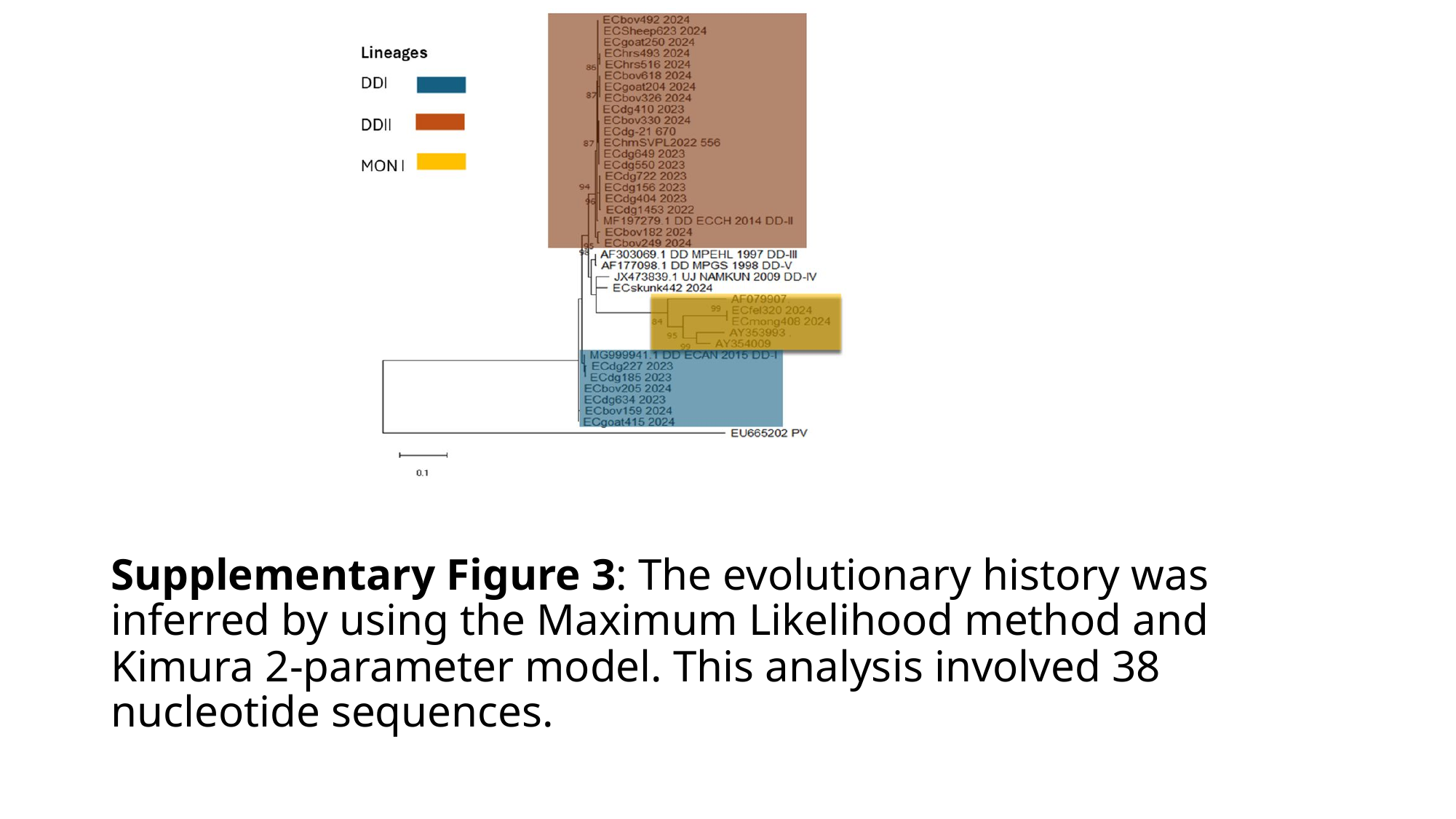

# Supplementary Figure 3: The evolutionary history was inferred by using the Maximum Likelihood method and Kimura 2-parameter model. This analysis involved 38 nucleotide sequences.
